# Benzodiazepine receptor agonist deprescribing principles for long-term use and dependence: modified Delphi recommendations from a multi-disciplinary expert panel

**DOI:** 10.1177/20451253261457547

**Published:** 2026-06-09

**Authors:** Jaden Brandt, Nicole Lamberson, Jolene Bressi, Alexis Ritvo, Janice Curle, Josef Witt-Doerring, Marjorie DeWert, Steven Wright, Mark Horowitz

**Affiliations:** Alliance for Benzodiazepine Best Practices, Portland, OR, USA; College of Pharmacy, Rady Faculty of Health Sciences, University of Manitoba, 750 McDermot Ave., Winnipeg, MB R3E 0T5, Canada; Benzodiazepine Information Coalition, Venice, FL, USA; Alliance for Benzodiazepine Best Practices, Portland, OR, USA; School of Public Health, Yale University, New Haven, CT, USA; Alliance for Benzodiazepine Best Practices, Portland, OR, USA; Psychiatry, University of Colorado Anschutz, Anschutz Medical Campus, Aurora, CO, USA; Benzodiazepine Information Coalition, Venice, FL, USA; Benzodiazepine Information Coalition, Venice, FL, USA; Alliance for Benzodiazepine Best Practices, Portland, OR, USA; Alliance for Benzodiazepine Best Practices, Portland, OR, USA; North East London NHS Foundation Trust, Research and Development, London, UK

**Keywords:** benzodiazepine, dependence, deprescribing, tapering, withdrawal

## Abstract

**Background:**

Benzodiazepine receptor agonists (BZRA) are frequently tapered because of risk of dependence and hazards of use in ageing. Standard tapering protocols may fail or result in harm from improper utilization of pharmacologic principles, lack of recognition of withdrawal and/or failures in shared decision-making.

**Objective:**

The aim of this work was to derive principles from experts to optimize deprescribing success while minimizing withdrawal among patients who use BZRA medication long-term.

**Design:**

Modified Delphi consensus study among experts from two not-for-profit organizations dedicated to BZRA use issues.

**Methods:**

Three stages of anonymized voting were conducted among clinical experts and patient representatives. An 80% agreement (‘agree’ or ‘strongly agree’ on a five item Likert-type scale) was required for consensus. Recommendations which failed to reach initial consensus, were modified from feedback and downgraded to ‘moderate’ (>80% agreement) or ‘weak’ consensus (50%–80% agreement) in the subsequent rounds.

**Results:**

A total of 35 of 48 invitees participated (73% response rate) which included seven family physicians, nine psychiatrists, five pharmacists, six patient advocates, two nurse practitioners, two licensed clinical social workers, two health service policy researchers, a physician assistant and a psychotherapist. A total of 31 of 35 participants were from the United States, with the remaining representatives from Canada, the UK and Ireland. Strong consensus was achieved for attaining informed consent prior to deprescribing (recommendation 1), using a flexible and gradual tapering approach (recommendation 2) characterized by shared decision-making (recommendation 3) with hyperbolic dose reductions (recommendation 4) facilitated via novel preparation techniques or compounded pharmaceutical formulations (recommendation 7). Reversion to previous doses may occur if necessary to reduce the incidence of withdrawal (recommendation 6). Strong consensus was also reached for adjunctive psychosocial interventions (recommendation 8) and/or peer-support resources (recommendation 9). Moderate and weak consensus was achieved, respectively, for step-wise conversion to a longer-acting BZRA (recommendation 5) and the avoidance of adjunctive non-BZRA pharmacotherapy (recommendation 10).

**Conclusion:**

This consensus guidance document for primary care providers, mental health clinicians and long-term users of BZRA outlines ten principles/recommendations intended for improving deprescribing outcomes with an emphasis on minimizing withdrawal risk.

## Plain language summary

Expert consensus on benzodiazepine tapering

Many individuals find significant difficulty in reducing their benzodiazepine or Z-Drug to discontinuation. This consensus study was administered and organized jointly by two not-for-profit organizations dedicated to benzodiazepine safety. A multi-round anonymous voting and feedback method was used which confirmed a high-degree of consensus among 8 out of 10 recommendation statements. The other two recommendation statements had moderate and low consensus, respectively. This work presents a practical framework for clinicians and patients to use for minimizing withdrawal related harm from benzodiazepines while ensuring gradual success toward discontinuation.

## Introduction

In the 1970s and 1980s, benzodiazepines (BZD) gained prominence in use for common anxiety and sleep complaints in medical practice.^1,2^ Z-Drugs such as zopiclone, zolpidem and zaleplon are structurally distinct molecules possessing similar pharmacology that were introduced in the 1990s as a supposedly safer option for insomnia over conventional benzodiazepines.^3^ Gradually, there was recognition that both of these drug classes (collectively referred to hereafter as ‘Benzodiazepine Receptor Agonists’ – BZRA) produced cognitive impairment and physical dependence that potentially outweighed any benefit from long-term use.^4–6^ Since at least the 1990s, a preponderance of guidelines have consistently advocated for ‘short-term’ use of these agents; typically for durations no longer than 4 weeks of continuous use.^7^ Despite observational evidence that prescribed use has declined in several middle to high-income countries from 2008 to 2018, usage has remained comparatively higher on a per-capita basis in Europe and North America.^8^ For instance, in 2018, BZRA usage ranged from 26.34 to 75.36 Defined Daily Doses per Thousand Inhabitants Per Day (DDD/TID) throughout Europe and North America, whereas Asia, Africa, Latin America and Oceania reported usage between only 5.23 and 27.22 DDD/TID.^8^

While BZRA indeed work rapidly and effectively for anxiety and insomnia, there are conflicting perspectives in the literature as to the merits and risks of long-term use.^9–11^ Potentially inappropriate maintenance prescribing of BZRA, because of an under-estimation of risk and an over-estimation of efficacy, is likely to result in tolerance, dependence and withdrawal.^12,13^ This clinical impression, shared by many patients and providers, as well as the well-established barriers to deprescribing, serve as compelling explanations for the ‘second’ prescription drug epidemic (after opioids) observed by others.^14–16^ However, a collective focus on deprescribing that has emerged in recent years raises a legitimate concern about a potential future epidemic, characterized by avoidable BZRA withdrawal harm from inappropriate deprescribing practices.

Historical taper protocols often proceeded rapidly at reduction rates ranging between 25% and 50% every 1–2 weeks and/or involved alternate-day dosing, which can be problematic due to significant fluctuations in drug exposure.^17^ While such reduction rates may be successful for many patients, it remains difficult to predict which individual may be inadvertently harmed via withdrawal at these reduction rates. Provocation of withdrawal via rapid reduction may precipitate ongoing symptoms that indicate neurobiologic processes that are reminiscent of ‘complex-persistent BZRA dependence’ or even ‘Benzodiazepine Induced Neurologic Dysfunction’; clinical constructs that remain controversial and not yet validated through neuropathology research but are nevertheless well conceptualized and supported by documented patient experience.^18,19^

**Figure 1.**
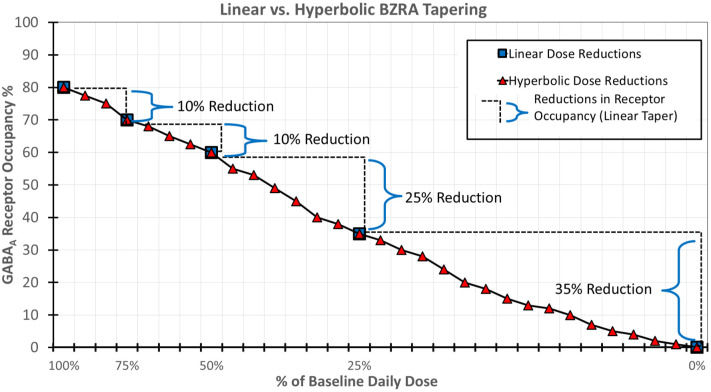
Hyperbolic versus linear gradual BZRA taper. Dose reductions are presented on a logarithmic *x*-axis. The traditional approach of linear dose reductions (squares) – 100% of dose, 75%, 50%, 25%, stop – produces increasingly large reductions in GABA receptor occupancy (from 10 percentage points for the first step to 35 percentage points for the last step), potentially associated with escalating withdrawal effects. By contrast, hyperbolic tapering (triangles) ensures that reductions are equally spaced in terms of receptor occupancy with the aim of minimizing withdrawal effects at the critical smaller doses. BZRA, benzodiazepine receptor agonists.

Since the U.S. Food and Drug Administration highlighted the potential for protracted or even permanent withdrawal symptoms in 2019, there is a growing consensus that tapering must be approached with greater caution.^20^ Hyperbolic tapering has been postulated as a rational pharmacologic approach wherein the implemented pattern of dose reductions more closely approximates reductions in target receptor binding occupancy.^21,22^ This more gradual tapering technique is hypothesized to limit the neurophysiologic withdrawal response that is prompted by the absence of the drug in a neuro-adaptational brain-state.^23,24^ Figure 1 depicts a comparison of the standard linear tapering method versus hyperbolic (logarithmic) tapering in terms of receptor occupancy (*y*-axis) and step-wise dose reductions (*x*-axis).

There are several deprescribing guidelines on BZRA for patients and providers to refer to.^17,25–27^ This work builds upon previous guidance by offering unique recommendations, from our collective experience and research, where higher quality evidence may be sparse but anecdotal real-world experience is plentiful. Therefore, some of these recommendations are weak in conventional evidence but strong in clinical rationale and/or experience, warranting further research or investigation for future improvement. For example, while randomized controlled trials comparing different tapering protocols are lacking, experiences of withdrawal and associated management strategies discussed in peer-support forums (with tens of thousands of registered users) have provided a compelling source of real-world evidence that is patient-driven.^19,28,29^ Furthermore, research priority setting partnership exercises have demonstrated a need for progressive guidance that aligns with patient experience while still acknowledging existing clinical uncertainties around psychotropic medication deprescribing.^30^

In general, the guidance outlined herein is intended to advise prescribing clinicians in their efforts to assist individuals who have expressed an interest or desire in discontinuing their BZRA medication and who, understanding the risk of withdrawal from conventional dose-reduction strategies, wish to reduce their dose at a rate that is less likely to provoke symptoms when there is otherwise no clear clinical urgency to taper more rapidly. It may be especially useful for those who have previously not found success in BZRA discontinuation from more rapid and/or protocolized dose tapering regimens. It is not intended for those who require a BZRA to control a seizure disorder, those with unstable or unmanaged substance use disorders, or for those in a mental health crisis where a BZRA is currently indicated.

## Methods

### Overview

A multi-stage consensus voting panel was held between clinician and patient representatives from the Alliance for Benzodiazepine Best Practices (ABBP) and the Benzodiazepine Information Coalition (BIC), as well as affiliated external experts. Both ABBP and BIC are non-profit organizations founded in the United States. While mission statements differ slightly, both organizations are similarly dedicated to improving BZRA prescribing and reducing associated harms. ABBP and BIC have jointly contributed towards this goal by educating patients and health professionals alike, producing resources for patient-provider encounters, advocating for clinical practice guideline revisions and engaging in participatory action research involving the patient community of those harmed by BZRA.^31^ Each organization has a board of directors complemented by a medical advisory board of clinicians and persons with lived experience. All representatives from both organizations were invited to participate in the voting panel.

For this project, two representatives from each organization (BIC – JC, NL/ABBP – JB, JB) along with an external consulting subject matter expert (MH), comprised a steering committee to oversee the staged progression of the consensus. The professional backgrounds for the steering committee were two clinical pharmacists (JB, JB), a physician assistant and BZRA tapering coach (NL), a patient advocate with lived experience (JC) and a psychiatrist with psychotropic deprescribing expertise (MH). The Maudsley Deprescribing Guidelines,^32^ the Ashton manual,^33^ a recent scoping review on benzodiazepine deprescribing guidance,^7^ a published textbook on BZD^34^ and a review article endorsed by the International Taskforce on Benzodiazepines^35^ served as the primary relevant background information for the initial approach to drafting the recommendations. These resources were identified and selected based on their notable relevance, comprehensiveness and the diversity of expert voices represented on the topic of BZRA. Other evidence was permitted to be brought forward by panellists as applicable. A systematic review was not undertaken for this project, but a systematic search of the literature was conducted previously as part of a scoping review project by members of the authorship team.^7^

Consensus was iteratively achieved through three stages of increasing participation by members of both organizations, following a modified Delphi process.^36^ Once an early informal consensus was reached, a brief, anonymous questionnaire containing all the position recommendations on a Likert scale (ranging from strongly disagree to strongly agree) was distributed to directors and advisors of both organizations. Those statements that achieved less than 80% combined agreement (strongly agree + agree) were discussed and brought back for a final stage of revision. The entire process, encompassing four stages (three consensus stages and one confirmation stage, all described below), occurred from May 1st to December 8th, 2024. This study was not pre-registered as a protocol, was not funded and did not offer any incentives to voting participants. The Qualtrics© web platform was used for survey creation, distribution and results collection at every stage of the project. This study is reported as per the ACCORD guidance statement for consensus research.^37^ (Supplemental Appendix 1) Details, including credentials, professional role, nationality, organizational affiliation and extent of participation for all individuals involved in this project are provided in Supplemental Appendix 2. The survey questionnaire is provided in Supplemental Appendix 3.

### Consensus stages

#### Stage 1 – Initial drafting of recommendations

The steering committee (JB, JB, NL, JC, MH) iteratively drafted, discussed and debated the nuances of the recommendations, virtually and via email, with revisions occurring until early informal consensus was reached. The recommendations were then uploaded by the project manager (JB) and disseminated via Qualtrics© as a piloting exercise to confirm the earliest consensus prior to formal voting stages. Once all five steering committee participants ‘strongly agreed’ to the initial draft recommendations, the project then commenced to the next stage.

#### Stage 2 – Small group survey

In stage 2, an anonymous survey was disseminated to the original five members of the steering committee, along with seven additional experts, selected to ensure an equal representation from both organizations as well as a mix of clinical and lived experience. At this stage, an 80% agreement threshold was required on each recommendation before proceeding to stage 3. A 14-day discussion period was held after consensus thresholds were reached to allow dissenters to state reasons for disagreement publicly to the group or to the project manager privately to be raised anonymously. Discrepancies and/or disagreements were resolved iteratively through either revision or survey re-distribution. Results were recorded and maintained on Qualtrics, with aggregate data shared via email to participants.

#### Stage 3 – Large group survey

Stage 3 involved the revised recommendations from stage 2 being disseminated to all advisors and board directors of both BIC and ABBP. This also included all participants from Stages 1 and 2 to allow for different voting if opinions had changed. A total of 48 invitations yielded survey responses from 33 participants (68% response rate), which included seven primary care physicians, nine psychiatrists, five pharmacists, six patient advocates, two health service policy researchers and one each for a physician assistant, nurse practitioner, psychotherapist and licensed clinical social worker. At this stage, an 80% agreement had to be reached on each recommendation before proceeding to finalization by the executive panel. When recommendations did not meet an 80% agreement threshold, anonymous text feedback from surveys was considered by the executive panel and, where suggestions were repeated (>2 mentions of the same problem), implemented into revised versions of the recommendations where sensible to do so. These changes were then circulated via a shortened survey (binary choice – agree vs disagree) back to the respondents for approval (Stage 3b).

#### Executive panel finalization of recommendations

The project manager/facilitator (JB), the medical directors for both organizations (AR, NL) and the consulting external psychotropic deprescribing expert (MH) met virtually to finalize the recommendations after independently reviewing all results and text feedback from stages 2 and 3. When minor changes to wording were suggested anonymously by the voting participants, without changing the content of the recommendations, these editorial suggestions were reviewed and implemented by the panel via informal agreement. Given the core background material (see overview above) and the knowledge of the broader literature by the executive panel, agreement was reached on the level of evidence applied to each recommendation using the Oxford Centre for Evidence-Based Medicine scale.^38^

### Strength of consensus and evidence grading

Consensus for each recommendation was categorized as ‘strong’, ‘moderate’ or ‘weak’ depending on the results. Those recommendations that achieved over 80% agreement at all stages were denoted as ‘strong’ consensus. Recommendations that proceeded to Stage 3 in spite of failure to reach 80% agreement were denoted as ‘moderate’ consensus if the revised version achieved >80% agreement and ‘weak’ consensus if the proportion of respondent agreement was between 50% and 80%. Evidence was graded independently by the executive panel members and then cross-verified according to the five-level scheme from the Oxford Centre for Evidence-Based Medicine as a confirmation process.^38^

## Results – Final recommendations

**Figure 2.**
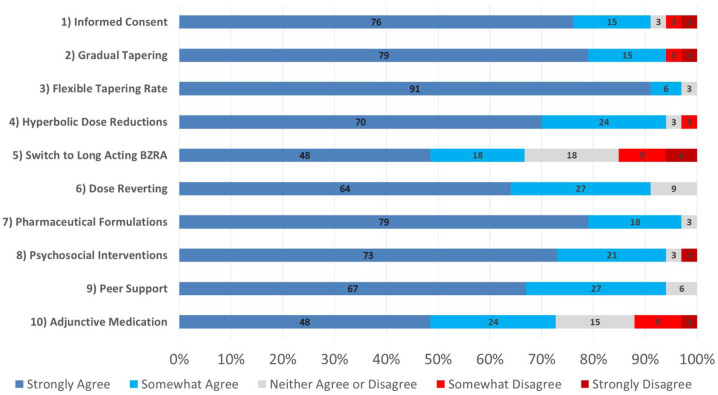
Extent of agreement per statement at final consensus (stage 3).

After four stages of consensus building, the following 10 principled statements were derived, eight with strong consensus, one with moderate consensus and one with weak consensus. Table 1 provides the final statements along with their evidence grade. The explanatory comments in Table 1 were written by the authors only (not voted on by panellists) and are included to qualify and contextualize each recommendation. The final consensus results are depicted in Figure 2 and correspond to the same order of statements presented in Table 1. Final tabulated results in the form of anonymized data are presented in Supplemental Appendix 4. Anonymized feedback comments, grouped by statement, are made available in Supplemental Appendix 5.

**Table 1.** Consensus statements and recommendations.

Recommendation/position statement	Explanatory comment
(1) Patients and/or caregivers should provide informed consent regarding deprescribing after receiving appropriate patient education about the most common signs and symptoms of withdrawal, along with warnings about withdrawal-induced akathisia and protracted withdrawal syndrome. **(Strong Consensus, Oxford Evidence Rating 5)**	Patient education is crucial for recognition of withdrawal symptoms and assists in self-management and earlier implementation of corrective mitigation strategies. Informed consent, occasionally in the form of signed-agreements ensure patients understand the risks involved in withdrawing from a BZRA.^39,40^
(2) Gradual dose tapering remains the primary foundational strategy underlying successful discontinuation of BZRA. For those for whom physical (physiologic) dependence has developed, this may mean a time course of several months and sometimes years before complete BZRA cessation. **(Strong Consensus, Oxford Evidence Rating 1)**	Research and decades of clinical observation have repeatedly demonstrated that abrupt cessation can produce significant withdrawal symptoms and may even endanger patients by causing seizures.^41–43^ Neuro-biologic adaptations, occurring from continued BZRA exposure, which manifest as drug tolerance and subsequent withdrawal syndromes, are most reliably managed through individualized gradual dose-reduction strategies.^44^
(3) The rate of gradual dose tapering should remain flexible and occur within a shared decision-making capacity, where the rate is guided by the patients’ withdrawal symptoms whenever possible. **(Strong Consensus, Oxford Evidence Rating 5)**	Paternalistic de-prescribing approaches may be harmful to patients and can damage the prescriber-patient relationship in terms of trust. Approaches to gradual dose tapering that recommend discontinuation within a specified time period may place undue pressure on patients to reduce the dose before they are prepared. An exceptional situation where prescribers may exercise more control over the tapering rate is in the presence of a legitimized substance use disorder, where the risk for either overdose or diversion may need to be actively managed by over-riding a patient’s stated desire for dose increases or a slower taper. However, BZRA use disorder is rare (0.5%–2.5%) but presents similarly to other substance use disorders and is characterized by craving and compulsive drug use.^45,46^ Even in the presence of addiction, forced tapers may result in relapse and access to BZRA obtained via illicit means. The right balance of patient harm-reduction with controlled access, such as stricter quantity limits and durations to limit drug diversion, is best managed by addiction medicine specialists and falls outside of the scope of this work.
(4) For BZRA users with physical (physiologic) dependency, it is prudent to reduce dosage by 10% or less (calculated on the previous dose so that reductions become smaller and smaller) per month up until discontinuation. Final doses before cessation may need to be as low as 1% of the original dose at the beginning of the taper. Some individuals may require even slower rates of reduction. Notwithstanding, patients who tolerate larger dose reductions or desire faster tapering may do so with careful supervision and monitoring. **(Strong Consensus, Oxford Evidence Rating 5)**	The law of mass action dictates that a hyperbolic pattern between dose and receptor occupancy exists for several classes of psychotropic drugs, including BZRA, which explains clinical response including withdrawal effects.^47–49^ An understanding of this phenomenon, as it relates to BZRA withdrawal, lends rational support to the probable superiority of hyperbolic dose reductions over linear dose reductions as a more comfortable tapering method towards BZRA discontinuation.
(5) Switching to a long-acting BZRA (particularly diazepam), in a step-wise fashion, could be considered for those patients with pronounced inter-dose withdrawal or those with difficulties tapering from a short-acting BZRA. An alternative approach is to dose a shorter-acting BZRA, already in use, more frequently. **(Moderate Consensus, Oxford Evidence Rating 1)**	Longer-acting BZRA (diazepam, clonazepam, and others), because of their extended half-life and/or active metabolites, have a lower risk of withdrawal than comparatively shorter-acting BZRA (alprazolam, lorazepam, triazolam, Z-Drugs). Evidence is mixed regarding the benefit of this strategy overall, but most evidence supports using diazepam when switching is chosen.^50–52^ Dose-equivalencies are approximate, and the potential risk for accumulation or over-sedation in older adults, those with hepatic impairment and/or potential drug-interactions, as well as under-dosing some patients, must be weighed carefully against any expected benefit to assist in discontinuing BZRA.
(6) Pausing the taper or reverting back to a previous dose should occur if withdrawal symptoms are intolerable and/or if the patient requests this according to their need to stabilize their symptoms for improved functioning. Subsequent tapering should then be made more gradual. **(Strong Consensus, Oxford Evidence Rating 5)**	Patient well-being should always be prioritized over any particular goal for achieving a lower dose or BZRA cessation. It is the case that not all patients are able to stop BZRA and a sense of failure or shame should never be perpetuated or imposed on patients because of this. Increasing back to a previous dose (e.g. going back one or more previous steps or to the original dose) is often a remedy for rapidly improving a patient’s quality of life. A more conservative dose-reduction strategy may then be pursued after a period of reflection and/or re-motivation.
(7) Techniques or pharmaceutical formulations to facilitate hyperbolic tapering,^a^ including micro-tapering,^b^ should be presented to patients and/or caregivers on an individualized basis according to their ease of understanding, acceptability for safe administration, product availability, cost and/or coverage. **(Strong Consensus, Oxford Evidence Rating 5)**	There is a lack of controlled trials of methods to taper BZRA (for both gradual hyperbolic and rapid linear tapers) at this time, gradual hyperbolic tapering has empirical support in other psychotropic drug classes and has been implemented successfully by thousands of patients within clinical practice and within patient-led community forums.^22,28,53^ Prescribers and pharmacists have a shared responsibility for guiding patients towards available compounded pharmaceutical formulations to facilitate accurate hyperbolic dose reduction.^54^ While availability and cost may be significant limiting factors in the practicality of this method, it is expected to be a preferred solution for many who desire BZRA discontinuation but also fear withdrawal symptoms or who have faced significant withdrawal challenges during past tapering attempts. Nevertheless, care must also be taken with liquid formulations in dose preparation where measurement error or misadministration may lead to acute harm.
(8) Psychosocial interventions should be offered and individualized according to the values, culture and needs of patients to assist in the BZRA discontinuation process. **(Strong Consensus, Oxford Evidence Rating 1)**	Existing evidence demonstrates the benefit of cognitive-behavioural therapy, particularly when used as a simultaneous treatment strategy for underlying conditions such as insomnia or anxiety disorders.^52,55–57^ However, we caution counsellors or motivational coaches to use a trauma-informed approach where appropriate and to resist strong emphasis on psychosomatic explanations for withdrawal symptoms. Psychosocial interventions alone should not be used as a substitute for gradual dose reduction, but could be a useful adjunct for many patients.
(9) Peer-support communities may be helpful for those undergoing BZRA withdrawal or tapering. **(Strong Consensus, Oxford Evidence Rating 4)**	People with a shared experience may relate to one another better on certain subjects. The internet has allowed people from disparate geographic locations to connect with one another in this way. Community forums for those taking psychotropic medication, such as benzobuddies.org and survivingantidepressants.org, have long offered individuals a place to provide and receive peer support, which may include encouragement and/or advice.^58^ However, occasions of negative experience stories, poor advice and/or incivility may still occur on the internet and patients should be reminded about this as a possibility despite our collective observations of generally positive experiences.^59^
(10) Adjunctive non-BZRA pharmacotherapies should only be considered as options of last resort given the scarcity of evidence and risk of associated adverse effects. **(Weak Consensus, Oxford Evidence Rating 1)**	Use of a medication from another drug class may create further complexities in a patient’s care or compromise their safety because of the adverse effects of their own. Even agents with the most positive cumulative evidence for assisting patients in BZRA discontinuation have shown conflicting results with low overall efficacy.^60–63^ The utility of the several agents that have been tried is controversial and should be limited to exceptional occasions or where a legitimate primary use for a particular medication is indicated and BZRA withdrawal management is a secondary purpose of convenience (i.e ‘dual’ indication).

a Refers to making smaller and smaller reductions as the dose gets lower, following the drug-binding curve. Implementation requires doses smaller than the lowest available solid dosage form, including manufacturer’s liquids (used as is or diluted); compounded liquids or mini-tablets, dispersions of tablets in water or other suspending vehicles; and tablet shaving.

b Micro-tapering, a sub-type of hyperbolic tapering, informally refers to a practice of making very small reductions more often (e.g. daily reductions), often requiring a liquid formulation of a drug, rather than larger reductions less often (e.g. often made every 2 or 4 weeks).

## Discussion

This consensus report builds on the topic of BZRA deprescribing by providing numerous insights, distinct from prior guidelines in several important ways. First, it is the only expert consensus panel, to our knowledge, that specifically focuses on patients who have been on these medications for long periods and may require more complex tapering strategies. By involving experts who routinely manage these extended-use cases, as well as individuals with lived experience of BZRA withdrawal, the recommendations prioritize patient realities and acknowledge the nuanced challenges of long-term dependence.

Second, the guidance recognizes and addresses the increasingly acknowledged risk of protracted withdrawal – an issue that was not widely discussed in earlier guidelines but has since been reviewed in detail by the recent American Society of Addiction Medicine guidelines on BZD tapering.^7,27^ By advocating for patient-led, symptom-guided, and sometimes slower taper rates, this consensus offers a more protective framework. Patients are thus better supported in preventing long-term complications, affirming the principle that prevention of harm should guide clinical decision-making around BZRA tapering.

Although all recommendations achieved consensus, recommendations 5 and 10 were only rated at moderate and weak consensus, respectively because of the need for their revision during stage 3. Step-wise substitutive tapering with a long-acting BZRA (diazepam in particular) presented some dissenting opinions among panellists based on the anonymized feedback comments (Supplemental Appendix 5). For instance, a few panellists supported the idea of earlier transition to diazepam, given their own success with this method and cited past difficulties with tapering a shorter-acting BZRA:

Panelist #24: ‘*I would not make the patient prove that they have inter-dose withdrawal before moving to diazepam. This choice could be part of the initial discussion with the patient, letting them decide whether to stick with the short-acting benzo or begin with a switch to diazepam*’.

Panelist #12: ‘*I did find significant relief, an evening out of withdrawal/tolerance symptoms when I was crossed over from alprazolam to diazepam using Ashton’s cross over schedule. While I know many have a hard time crossing to diazepam, I found it very beneficial* . . .’

Other panellists presented alternative viewpoints that maintained the risks of switching to a long-acting BZRA due to improper consideration of patient-drug differences in pharmacokinetics and variable cross-tolerance:

Panelist #6: ‘*Switching from one BZD to another should be avoided as new substances may affect each patient differently. Providers should encourage patients to remain on their current BZD which may require more frequent dosing to reduce possibility of complications from using an alternative BZD*’.

Regarding adjunctive non-BZRA pharmacotherapies, the consensus was even more divided in general opinion about these various medications. Some panellists felt that the initiation of new medication may result in further risk or new clinical problems for individuals already experiencing BZRA withdrawal:

Panelist #4: ‘*Add-on medications and supplements are extremely risky for those with impaired central nervous systems from benzodiazepine exposure*’.

Panelist #11: ‘*. . . Providers typically do not appreciate the prevalence of adverse effects in this population*’.

In contrast, other panellists saw value in the selection of adjunctive pharmacotherapies to target certain symptomatic presentations of BZRA withdrawal in the absence of known contraindications:

Panelist #24: ‘*I don*’*t see much downside with offering routine second-generation antidepressants approved for anxiety disorders or offering prn*’*s of meds like hydroxyzine. Barring cardiovascular contraindications, some patients may benefit from sympatholytics such as propranolol or clonidine, either routine or prn. Otherwise, the patient*’*s anxiety may be exacerbated if they feel they have no backup/‘failsafe’ to take, adding to the anxiety caused by benzo [withdrawal]*’

In both cases, for recommendations 5 and 10, consensus was eventually achieved, at the subsequent stage and beyond the 80% threshold, by modifying the recommendation wording from being very conservative (i.e from ‘only be used in cases. . .’ in rec. 5 initial) to moderate in its prescriptiveness (i.e to ‘could be considered. . .’ in rec. 5 final) allowing greater clinical discretion albeit with caution still emphasized.

### Limitations

Although this consensus statement benefits from broad stakeholder engagement – integrating clinical, research, and patient lived experience perspectives – it has some limitations. Firstly, the iterative nature of the consensus process may reflect the views of a self-selecting group closely involved in BZRA withdrawal issues, which may limit the generalizability to a wider population of prescribers or patients. Furthermore, the consensus threshold of 80% agreement – while commonly employed – does not rule out the possibility that minority dissenting opinions may offer important or novel insights not fully captured in the final recommendations despite attempts to do so via the survey feedback and review process. An additional limitation was that the survey construction and pilot testing were only conducted among the steering committee members and not formally validated.

A rigorous systematic review and integrated evidence-rating approach, such as GRADE, was also not taken for this project, but has been done previously in other deprescribing guidelines.^17,64^ However, our intent was not to produce a comprehensive guideline but rather to derive a joint position deprescribing consensus document endorsed by both BIC and ABBP.

It is also probable that these recommendations lean towards the conservative side, as many participants either have direct lived experience of harm or treat complex BZRA-related cases. The recommendations themselves, particularly numbers 4 and 7, which correspond to the process of hyperbolic tapering, may also be particularly challenging for implementation in routine primary care practice, given constraints arising from commercially available standard BZRA products from pharmacies and poorer availability of compounded alternatives.^65^ A recent systematic review has demonstrated that there is very limited research available on dosage form manipulation for tapering psychotropic medications.^66^ In regards to future policy and best practice, clinical approaches may need to evolve to incorporate earlier implementation of hyperbolic tapering by modifying existing prescription product selection in both physician prescribing and community pharmacy dispensing software to begin normalizing the use of certain compounded BZRA for the purpose of hyperbolic tapering (e.g lorazepam 0.1 mg/mL). Pharmacies that dispense these products for this specific purpose may also consider developing or utilizing standardized information/educational handouts given the unique nature and safety considerations for BZRA in liquid form (risk of ingestion error, differences in rate and extent of absorption).

### Contradictory research findings

The reliance on anecdotal and patient-reported experiences, while highly relevant to real-world practice, may introduce subjective biases that differ from evidence generated by more controlled study designs. As such, our recommendations should also be justified in consideration of some recent, potentially contradictory, research findings.

An observational study from Maust et al. found that BZRA discontinuation may actually be associated with a small, 2%–2.5% absolute risk-difference increase in mortality compared to those who remain on their BZRA.^67^ This surprising finding contradicts previous studies that had implicated BZRA use with increasing mortality.^68,69^ One untested hypothesis could be that more rapid linear or abrupt tapers provoke withdrawal-induced physiologic stress, which may result in a small absolute increase in mortality among vulnerable individuals. As such, it remains a possibility that both long-term BZRA use as well as abrupt or rapid linear tapers (i.e. acute, iatrogenic BZRA withdrawal) may increase mortality risk, albeit through different mechanisms of harm. Therefore, given the known and potential harms of BZRA, it remains a reasonable goal for many to attempt gradual reductions, in a manner which minimizes withdrawal, towards eventual discontinuation.

Another study that warrants mention, in the context of our recommendations, is that of Fung et al.^70^ This was a randomized clinical trial (*n* = 188) which demonstrated higher success with a masked (i.e blinded) taper compared to an open-label taper in achieving BZRA discontinuation.^70^ The possibility of ‘cognitive expectancy’ (i.e nocebo effects) playing a role in the emergence of withdrawal symptoms was evoked as a plausible explanation. However, the ethical and practical complications of ‘blinding’ patients in routine clinical practice undermine this approach. It is also the case that their study may not be generalizable given the comparatively lower daily doses used at baseline (⩽8 diazepam milligram equivalents). In contrast, the principles outlined here, grounded in a patient care philosophy of shared decision-making and harm minimization, are expected to alleviate anxiety about withdrawal, reduce withdrawal itself and facilitate patient empowerment despite a lengthier tapering course, which may be contrary to some prescribers’ own goals for their patients (i.e paternalism).

### Future directions

A significant barrier to investigating BZRA deprescribing is the limitation of standard clinical trial designs. Early-phase studies for new drugs typically last for only a few weeks or months, a duration insufficient for patients to develop substantial physical dependence. As a result, withdrawal outcomes observed over such short periods may not generalize to individuals who have been on BZRA for years or even decades. This gap highlights the need for alternative research approaches that capture the complexities of long-term use and withdrawal. Identifying patient-specific factors – be they genetic, metabolic, medical or psychosocial – that predict a need for a slower taper is another key area of inquiry, as is determining how variables such as duration of use or medication dose influence the ideal taper length. It may be possible to address these knowledge gaps through observational or prospective cohort studies designed around flexible tapering guidelines. Such designs would allow researchers to capture real-world patient outcomes while still gathering rigorous data on the effectiveness and safety of various taper speeds. By contrast, imposing strict taper protocols on randomized study participants – regardless of individual withdrawal responses – raises ethical concerns, given the potential for severe side effects ranging from mood instability to suicidal ideation. Innovations such as pharmacogenetic testing, serum concentration monitoring and withdrawal risk-score prediction may further assist in improving BZRA discontinuation.^71–73^ Future research should explore the safety, feasibility, and efficacy of various deprescribing strategies to strengthen guidance and improve patient outcomes.

## Conclusion

Substantial consensus exists around BZRA discontinuation strategies among prescribers, allied health professionals and patient advocate leaders associated with not-for-profit organizations and community forums dedicated to the topic of BZRA. This consensus document with 10 principled recommendations from experts should serve as a guide for improving clinical practice towards safer BZRA deprescribing, especially for long-term users of BZRA and/or those who have not been able to discontinue BZRA with conventional linear tapering strategies.

## Supplemental Material

sj-docx-1-tpp-10.1177_20451253261457547 – Supplemental material for Benzodiazepine receptor agonist deprescribing principles for long-term use and dependence: modified Delphi recommendations from a multi-disciplinary expert panel Supplemental material, sj-docx-1-tpp-10.1177_20451253261457547 for Benzodiazepine receptor agonist deprescribing principles for long-term use and dependence: modified Delphi recommendations from a multi-disciplinary expert panel by Jaden Brandt, Nicole Lamberson, Jolene Bressi, Alexis Ritvo, Janice Curle, Josef Witt-Doerring, Marjorie DeWert, Steven Wright and Mark Horowitz in Therapeutic Advances in Psychopharmacology

sj-docx-2-tpp-10.1177_20451253261457547 – Supplemental material for Benzodiazepine receptor agonist deprescribing principles for long-term use and dependence: modified Delphi recommendations from a multi-disciplinary expert panel Supplemental material, sj-docx-2-tpp-10.1177_20451253261457547 for Benzodiazepine receptor agonist deprescribing principles for long-term use and dependence: modified Delphi recommendations from a multi-disciplinary expert panel by Jaden Brandt, Nicole Lamberson, Jolene Bressi, Alexis Ritvo, Janice Curle, Josef Witt-Doerring, Marjorie DeWert, Steven Wright and Mark Horowitz in Therapeutic Advances in Psychopharmacology

sj-docx-3-tpp-10.1177_20451253261457547 – Supplemental material for Benzodiazepine receptor agonist deprescribing principles for long-term use and dependence: modified Delphi recommendations from a multi-disciplinary expert panel Supplemental material, sj-docx-3-tpp-10.1177_20451253261457547 for Benzodiazepine receptor agonist deprescribing principles for long-term use and dependence: modified Delphi recommendations from a multi-disciplinary expert panel by Jaden Brandt, Nicole Lamberson, Jolene Bressi, Alexis Ritvo, Janice Curle, Josef Witt-Doerring, Marjorie DeWert, Steven Wright and Mark Horowitz in Therapeutic Advances in Psychopharmacology

sj-pdf-5-tpp-10.1177_20451253261457547 – Supplemental material for Benzodiazepine receptor agonist deprescribing principles for long-term use and dependence: modified Delphi recommendations from a multi-disciplinary expert panel Supplemental material, sj-pdf-5-tpp-10.1177_20451253261457547 for Benzodiazepine receptor agonist deprescribing principles for long-term use and dependence: modified Delphi recommendations from a multi-disciplinary expert panel by Jaden Brandt, Nicole Lamberson, Jolene Bressi, Alexis Ritvo, Janice Curle, Josef Witt-Doerring, Marjorie DeWert, Steven Wright and Mark Horowitz in Therapeutic Advances in Psychopharmacology

sj-xlsx-4-tpp-10.1177_20451253261457547 – Supplemental material for Benzodiazepine receptor agonist deprescribing principles for long-term use and dependence: modified Delphi recommendations from a multi-disciplinary expert panel Supplemental material, sj-xlsx-4-tpp-10.1177_20451253261457547 for Benzodiazepine receptor agonist deprescribing principles for long-term use and dependence: modified Delphi recommendations from a multi-disciplinary expert panel by Jaden Brandt, Nicole Lamberson, Jolene Bressi, Alexis Ritvo, Janice Curle, Josef Witt-Doerring, Marjorie DeWert, Steven Wright and Mark Horowitz in Therapeutic Advances in Psychopharmacology

## Declarations

**Ethical approval and consent to participate:** This study was approved by a steering committee comprised of leadership from both the ABBP and the BIC. All survey respondents consented to participate and were solicited via email outreach by members of the steering committee. Individual data from survey responses remained anonymous. As this study was a joint-organizational endeavour, ethical approval from an institutional review board or research ethics board was not sought.

**Consent for publication:** All panellists/participants provided consent for publication verbally or in writing (email) to members of the steering committee at the time of initial survey dissemination and/or completion, in the understanding that this work was for a research publication endorsed by both the ABBP and the BIC. All panellists/participants involvement with either organization is detailed publicly on the organizational websites at either www.benzoreform.org or www.benzoinfo.com.

**Availability of data and materials:** Jaden Brandt is the guarantor of the study and can provide research data from Qualtrics upon reasonable request.
