# Supplementary material for: Benzodiazepine receptor agonist deprescribing principles for long-term use and dependence: modified Delphi recommendations from a multi-disciplinary expert panel
Source: Ther Adv Psychopharmacol. 2026 Jun 9;16:20451253261457547. doi: 10.1177/20451253261457547 (PMC13254137; doi:10.1177/20451253261457547)
Supplement: sj-docx-2-tpp-10.1177_20451253261457547 – Supplemental material for Benzodiazepine receptor agonist deprescribing principles for long-term use and dependence: modified Delphi recommendations from a multi-disciplinary expert panel [file sj-docx-2-tpp-10.1177_20451253261457547.docx]

| Name  (Degrees / Qualifications) | Professional Background | Nationality | Project Execution Role  *(if applicable)* | Relevant Organizational Affiliation and Role |  | Consensus Stage Participation | | | | |
| --- | --- | --- | --- | --- | --- | --- | --- | --- | --- | --- |
|  |  |  |  |  | Pre-Survey Draft | Stage 1 | Stage 2 | Stage 3 | | Recommendation  Finalization |
| Mr. Jaden Brandt (BSc.Pharm, MSc.) | Pharmacy | Canada | Project Manager, Steering Committee,  Executive Panel, Study Guarantor | ABBP, Board Director | **✔** | **✔** | **✔** |  | **✔** | **✔** |
| Dr. Mark Horowitz  (MBBS, PhD) | Psychiatry | United Kingdom | Steering Committee, Executive Panel | Invited External Psychotropic Tapering Expert | **✔** | **✔** | **✔** |  | **✔** | **✔** |
| Ms. Nicole Lamberson (MPA) | Physician Assistant | United States | Steering Committee  Executive Panel, | BIC, Medical Director | **✔** | **✔** | **✔** |  | **✔** | **✔** |
| Ms. Janice Curle | Patient Advocate / Representative | United States | Steering Committee | BIC, Co-Founder, Board Director | **✔** | **✔** | **✔** |  | **✔** |  |
| Dr. Jolene Bressi (Pharm.D) | Pharmacy | United States | Steering Committee | ABBP,  Board Director | **✔** | **✔** | **✔** |  | **✔** |  |
| Dr. Alexis Ritvo  (MD, MPH, BA) | Psychiatry | United States | Executive Panel | ABBP, Medical Director |  | **✔** | **✔** |  |  | **✔** |
| Dr. Josef Witt-Doerring  (MD) | Psychiatry | United States | N/A | ABBP & BIC, Advisory Board Member |  | **✔** | **✔** |  | **✔** |  |
| Dr. Steven Wright  (MD) | Medicine / Addiction Medicine | United States | N/A | ABBP,  Past Medical Director |  | **✔** | **✔** |  | **✔** |  |
| Dr. James Wright  (MD, Ph.D, FRCPC) | Medicine | Canada | N/A | BIC, Advisory Board Member |  | **✔** | **✔** |  | **✔** |  |
| Dr. Marjorie DeWert  (Ph.D) | Patient Advocate / Representative | United States | N/A | Observer / Participant Online Peer-Support Communities |  | **✔** | **✔** |  | **✔** |  |
| Ms. Gail Dawson  (BSc. MS) | Nursing | United States | N/A | BIC, Advisory Board Member |  | **✔** |  |  |  |  |
| Mr. Chris Paige  (LCSW) | Clinical Social Work | United States | N/A | BIC, Advisory Board Member |  | **✔** |  |  |  |  |
| Dr. Cathal Cadogan  (BSc.Pharm, Ph.D) | Pharmacy | Ireland | N/A | ABBP,  Board Director |  |  | **✔** |  | **✔** |  |
| Ms. Elizabeth McCarthy  (MA) | Psychotherapy | United States | N/A | BIC, Advisory Board Member |  |  | **✔** |  | **✔** |  |
| Dr. Chistopher Blazes  (MD) | Psychiatry | United States | N/A | ABBP, Board Director |  |  | **✔** |  | **✔** |  |
| Dr. Michael Bohan  (MD) | Medicine / Addiction Medicine | United States | N/A | ABBP, BIC  Advisory Board Member |  |  | **✔** |  | **✔** |  |
| Dr. Arwen Podesta  (MD) | Psychiatry / Addiction Medicine | United States | N/A | ABBP, Advisory Board Member |  |  | **✔** |  | **✔** |  |
| Dr. Seema Gupta  (MD) | Medicine | United States | N/A | BIC, Advisory Board Member |  |  | **✔** |  | **✔** |  |
| Dr. Akhil Anand  (MD) | Psychiatry | United States | N/A | ABBP, Advisory Board Member |  |  | **✔** |  | **✔** |  |
| Dr. Mark Leeds  (DO) | Medicine | United States | N/A | BIC, Advisory Board Member |  |  | **✔** |  | **✔** |  |
| Dr. Lori Mor  (Pharm.D) | Pharmacy | United States | N/A | ABBP,  Board Director |  |  | **✔** |  |  |  |
| Dr. Marissa Witt-Doerring  (MD) | Psychiatry | United States | N/A | ABBP, Advisory Board Member |  |  | **✔** |  |  |  |
| Mr. John Staight  (BA) | Patient Advocate / Representative | United States | N/A | BIC, Advisory Board Member |  |  | **✔** |  |  |  |
| Dr. Barbara Connolly  (Ph.D) | Patient Advocate / Representative | United States | N/A | BIC, Advisory Board Member  Benzo Warrior Senior Admin |  |  | **✔** |  |  |  |
| Dr. Jessica Angleson  (Pharm.D, MBA, BCPS) | Pharmacy | United States | N/A | ABBP, Advisory Board Member |  |  | **✔** |  |  |  |
| Mr. Bernard Silvernail  (MS) | Patient Advocate / Representative | United States | N/A | ABBP, Founder & President |  |  | **✔** |  |  |  |
| Ms. Terri Schreiber | Public Health / Health Services | United States | N/A | Schreiber Research Group |  |  | **✔** |  |  |  |
| Ms. Jezel Rosa  (MSN, APRN,  PMHNP-BC) | Mental Health Nurse Practitioner | United States | N/A | ABBP, Advisory Board Member |  |  | **✔** |  |  |  |
| Dr. Stephen LaCorte  (JD) | Patient Advocate / Representative | United States | N/A | BIC, Board Director |  |  | **✔** |  |  |  |
| Dr. Valsa Medhava  (MD, MPH) | Medicine / Addiction Medicine | United States | N/A | ABBP, Advisory Board Member |  |  | **✔** |  |  |  |
| Dr. Jayne Violette  (Ph.D) | Patient Advocate / Representative | United States | N/A | ABBP, Advisory Board Member |  |  | **✔** |  |  |  |
| Ms. Sonja Styblo  (LMSW) | Clinical Social Work | United States | N/A | BIC, Advisory Board Member |  |  | **✔** |  |  |  |
| Dr. Erick Turner  (MD) | Psychiatry | United States | N/A | ABBP, Advisory Board Member |  |  | **✔** |  |  |  |
| Dr. John Umhau  (MD, MPH) | Medicine | United States | N/A | ABBP, Advisory Board Member |  |  | **✔** |  |  |  |
| Dr. Tim MacDonald  (MBBS, FRANZP) | Psychiatry | United States | N/A | ABBP, Advisory Board Member |  |  | **✔** |  |  |  |
