# Supplementary material for: Benzodiazepine receptor agonist deprescribing principles for long-term use and dependence: modified Delphi recommendations from a multi-disciplinary expert panel
Source: Ther Adv Psychopharmacol. 2026 Jun 9;16:20451253261457547. doi: 10.1177/20451253261457547 (PMC13254137; doi:10.1177/20451253261457547)
Supplement: sj-docx-3-tpp-10.1177_20451253261457547 – Supplemental material for Benzodiazepine receptor agonist deprescribing principles for long-term use and dependence: modified Delphi recommendations from a multi-disciplinary expert panel [file sj-docx-3-tpp-10.1177_20451253261457547.docx]

Supplemental Appendix 3:

SURVEY - BIC/ABBP Consensus Statement on Deprescribing BZRA (Phase 3)

Start of Block: Default Question Block

Q1 **Recommendation 1** Patients and/or caregivers should provide informed consent after receiving appropriate patient education about the most common signs and symptoms of withdrawal along with warnings about withdrawal-induced akathisia and protracted withdrawal syndrome.

- Strongly Disagree (1)
- Somewhat disagree (2)
- Neither agree nor disagree (3)
- Somewhat agree (4)
- Strongly agree (5)

Q1-Comment Optional Comment/Feedback - Recommendation 1

________________________________________________________________

| Page Break |  |
| --- | --- |

Q2 **Recommendation 2** Gradual dose tapering remains the primary foundational strategy underlying successful discontinuation of BZRA – for those for which physical dependence has developed this may mean a time course of several months and sometimes years before complete BZRA cessation.

- Strongly Disagree (1)
- Somewhat disagree (2)
- Neither agree nor disagree (3)
- Somewhat agree (4)
- Strongly agree (5)

Q2 - Comment Optional Comment/Feedback- Recommendation 2

________________________________________________________________

| Page Break |  |
| --- | --- |

Q3 **Recommendation 3** The rate of gradual dose tapering should remain flexible and occur within a shared-decision making capacity, where the rate is led by the patients’ withdrawal symptoms whenever possible.

- Strongly Disagree (1)
- Somewhat disagree (2)
- Neither agree nor disagree (3)
- Somewhat agree (4)
- Strongly agree (5)

Q3 - Comment Optional Comment/Feedback - Recommendation 3

________________________________________________________________

| Page Break |  |
| --- | --- |

Q4 **Recommendation 4** For BZRA users with physiologic dependency, it is prudent to reduce dosage by 10% or less (calculated on the previous dose so that reductions become smaller and smaller) per month up until discontinuation. Final doses before cessation may need to be as low as 1% of the original dose at the beginning of the taper. Some individuals may require even slower rates of reduction. Notwithstanding, patients that tolerate larger dose reductions or desire faster tapering may do so with careful supervision and monitoring

- Strongly Disagree (1)
- Somewhat disagree (2)
- Neither agree nor disagree (3)
- Somewhat agree (4)
- Strongly agree (5)

Q4 - Comment Optional Comment/Feedback- Recommendation 4

________________________________________________________________

| Page Break |  |
| --- | --- |

Q5 **Recommendation 5** Switching to a long-acting BZRA (particularly diazepam), in a step-wise fashion, should only be considered for those patients with pronounced inter-dose withdrawal or those with difficulties tapering from a short-acting BZRA. An alternative approach is to dose a shorter acting BZRA, already in use, more frequently.

- Strongly Disagree (1)
- Somewhat disagree (2)
- Neither agree nor disagree (3)
- Somewhat agree (4)
- Strongly agree (5)

Q5 - Comment Optional Comment/Feedback - Recommendation 5

________________________________________________________________

| Page Break |  |
| --- | --- |

Q6 **Recommendation 6** Pausing the taper or reverting back to a previous dose should occur if withdrawal symptoms are intolerable and/or if the patient requests this according to their need to stabilize their symptoms for improved functioning. Subsequent tapering should then be made more gradual.

- Strongly Disagree (1)
- Somewhat disagree (2)
- Neither agree nor disagree (3)
- Somewhat agree (4)
- Strongly agree (5)

Q6 - Comment Optional Comment/Feedback - Recommendation 6

________________________________________________________________

| Page Break |  |
| --- | --- |

Q7 **Recommendation 7** Techniques or pharmaceutical formulations to facilitate hyperbolic micro-tapering should be presented to patients and/or caregivers on an individualized basis according to their ease of understanding, acceptability for safe administration, product availability, cost and/or coverage.

- Strongly Disagree (1)
- Somewhat disagree (2)
- Neither agree nor disagree (3)
- Somewhat agree (4)
- Strongly agree (5)

Q7 - Comment Optional Comment/Feedback- Recommendation 7

________________________________________________________________

| Page Break |  |
| --- | --- |

Q8 **Recommendation 8** Psychosocial interventions should be offered and individualized according to the values, culture and needs of patients to assist in the BZRA discontinuation process.

- Strongly Disagree (1)
- Somewhat disagree (2)
- Neither agree nor disagree (3)
- Somewhat agree (4)
- Strongly agree (5)

Q8 - Comment Optional Comment/Feedback - Recommendation 8

________________________________________________________________

| Page Break |  |
| --- | --- |

Q9 **Recommendation 9** Peer-support communities may be helpful for those undergoing BZRA withdrawal or tapering.

- Strongly Disagree (1)
- Somewhat disagree (2)
- Neither agree nor disagree (3)
- Somewhat agree (4)
- Strongly agree (5)

Q9 - Comment Optional Comment/Feedback - Recommendation 9

________________________________________________________________

| Page Break |  |
| --- | --- |

Q10 **Recommendation 10** Adjunctive non-BZRA pharmacotherapies are not routinely recommended given scarcity of evidence and risk of associated adverse effects.

- Strongly Disagree (1)
- Somewhat disagree (2)
- Neither agree nor disagree (3)
- Somewhat agree (4)
- Strongly agree (5)

Q10 - Comment Optional Comment/Feedback- Recommendation 10

________________________________________________________________

End of Block: Default Question Block
