# Supplementary material for: Benzodiazepine receptor agonist deprescribing principles for long-term use and dependence: modified Delphi recommendations from a multi-disciplinary expert panel
Source: Ther Adv Psychopharmacol. 2026 Jun 9;16:20451253261457547. doi: 10.1177/20451253261457547 (PMC13254137; doi:10.1177/20451253261457547)
Supplement: sj-pdf-5-tpp-10.1177_20451253261457547 – Supplemental material for Benzodiazepine receptor agonist deprescribing principles for long-term use and dependence: modified Delphi recommendations from a multi-disciplinary expert panel [file sj-pdf-5-tpp-10.1177_20451253261457547.pdf]

SURVEY - BIC/ABBP Consensus Statement on Deprescribing BZRA (Phase 3) / Page 1

Filters

Responses: 24

Optional Comment/Feedback - Recommendation 1 14

Optional Comment/Feedback - Recommendation 1

i think benefits and risk need to be tailored appropriately according to the situation. if someone is in an acute and highly distressed state where a BZD could help relieve symptoms of anxiety/insomnia, a very extensive discussions about things like akathisia may not be appropriate if only being prescribed for short duration

---

I strongly agree that patients/caregivers should ONLY be administered benzodiazepines for long term use (more than 1 week) after providing their informed consent after receiving appropriate patient education concerning the above risks. Whether patients/caregivers should provide their informed consent depends on the specific details of the patient's condition.

Caregivers provide both patient education and the basis for informed consent. Why single out akathisia? Reword: Caregivers should provide informed consent after delivering appropriate patient education about the most common signs and symptoms of withdrawal and protracted withdrawal syndrome.

I strongly agree. Ideally, this would allow the patient to opt out of the cessation process and continue using the medication, with dosage adjustments as tolerance develops.

Please note that there has been strong resistance to this concept amount providers in MA.

Patients and/or caregivers should provide informed consent after receiving appropriate patient education about the most common signs and symptoms of withdrawal along with warnings about the possibility of developing more severe issues including, but not limited to, withdrawal-induced akathisia and protracted withdrawal syndrome.

Without clarifying the risk of withdrawal induced akathisia or protracted withdrawal with quantitative risk estimates, this may simply dissuade people from ever trying to taper.

Is 'protracted withdrawal syndrome' known and recognized by clinicians? If not, it might be better to use plain language. For example, patients/caregivers should be warned that in some cases patients can experience post-cessation symptoms that may be both debilitating and long-lasting.

The context to this is not given. I think it means 'informed consent to deprescribing'? Along with this they should also be told about the likely harms of long term use of benzos to be able to make an informed choice about continuing or stopping

---

Informed consent should be integrated into physician and pharmacist workflows to enable better patient understanding of long term harms that can occur from taking benzodiazepines. Most are unaware that these harms can occur even after only brief exposure periods.

---

to include black boxed warnings

---

a link to a video with testimony from lay people might reach more people

---

Wording thoughts: perhaps state that consent should be given prior to prescribing. Why highlight akathiisia? Maybe "the range of physical and psychological symptoms including ..."

The informed consent should be more than some printed, densely worded statement. The prescriber should take their time really explaining the potential implications.

Optional Comment/Feedback- Recommendation 2 9

Optional Comment/Feedback- Recommendation 2

Patients should be educated on reliable methods of very gradual dose tapering, including the use of compounded liquid preps

---

Throughout: I think we were going to put physiological/physical - since FDA uses one and ASAM uses another?

---

with caveats.. holding etc.

I am not convinced that this is the best strategy. People that I observe that took this approach remain in BIND for many months or years.

Daily microtaper is safest way

This does not address two key points - the pace of a taper should be guided by the patient's response to reductions and reductions should get smaller and smaller as the dose gets lower.

Maybe worth here or elsewhere putting in a definition of what physical dependence is as compared to addiction. Perhaps provide a reason for why so long 'so that withdrawal symptoms are of a tolerable level. The slower people taper the lesser their withdrawal effects tend to be. This allows time for adaptations to the presence of the drug to resolve'

Looping this back to informed consent. Patient's are generally not aware that tapering can be such a long and tedious process. Given this information, it is my belief that many would choose to not resort to BZD's had they understood the intricacies of tapering.

AShton Manual and Maudsley Deprescribing text recommended references

---

Optional Comment/Feedback - Recommendation 3 8

Optional Comment/Feedback - Recommendation 3

"determined by" instead of "led by"? It is actually a balance between functionality and symptoms.

For those who are already physically dependent, the patient should have the option to discontinue tapering and choose to stay on the drug long-term if they prefer.

This recommendation should be coupled with better distinguishing of who has SUD vs. physical dependence as the SUD diagnosis gets very overused and misapplied.

I would consider revising to state "where the rate is led by the intensity of and tolerance for the patient's withdrawal symptoms."

Perhaps use 'guided by' instead of 'led by'

UNless there is some urgency to do with risky behaviour patient-led tapers tend to be more successful than dictated regimens.

Nothing in medicine should be a one size fits all. Having patient buy in through shared decision making and informed consent ensures that patients are willing and able to stick to long and oftentimes grueling taping protocols.

Prescribers need to be aware of hyperbolic principles in tapering (Maudsley Deprescribing Guidlines). Ashton Manual taper schedules end at 1 mg. Prescribers need to be prepared to taper below 1 mg (microtaper).aze Aston Manual does not addres

---

Optional Comment/Feedback- Recommendation 4 10

Optional Comment/Feedback- Recommendation 4

while this makes sense, I would like to see more robust evidence - especially in terms of whether this is required for all patients or just a subgroup

Some patients might not need this. But it should be the standard recommendation

I feel that this rate of reduction approximates to a reverse homeopathy approach and may keep people on BZD far longer than is necessary. I feel that removing the part in brackets (calculated on the previous dose etc.) will make this simpler and more practical while still being an improvement over current guidelines.

previous questions I may not answered the way I wanted to go- could not go back to review

I am not sure about this. I know many people believe this, but I am not 100% sure it is true. I would suggest a RCT study to validate this strategy

Perhaps a quick rationale 'because of the hyperbolic pattern of effect between dose and effect on target receptors, exponential/hyperbolic dose reductions are pharmacologically sensible'

Even with physiological dependence, not all BZRA users require slow tapers. Please don't put that this is the rule. In my experience it is the exception.

It is generally considered good practice throughout the pharmacy practice to start low and go slow. This is true not only of starting new therapies but also with tapering of old therapies. It is especially true when discussing tapering of benzodiazepines where GABA receptors have been down regulated due to drug exposure.

achieving such low fractions is not practical for most benzodiazepines, eg with clonazepam, the lowest strength tab is 0.5 mg. By contrast, diazepam allows greater flexibility.

Per month or longer

Optional Comment/Feedback - Recommendation 5 15

Optional Comment/Feedback - Recommendation 5

again, i would like to see the evidence - especially around the suggested alternative approach

This is dependent on taper method. The only methods that have literature evidence are pill cutting (to 1/4), uncut solutions, and compounding pharmacies. Using scales, solutions and suspensions have no medical studies backing them. Diazepam not only is longer-acting, but also comes in more formulations.

Diazepam works well for some people but it can cause significant issues for others, including intolerance and increased depression or suicidal ideation.

Sometimes patients don't have access to compounding and struggle with weighing pills or making a water taper; for these people diazepam may be a more practical solution

inclined to agree but beyond my expertise to feel 100% confident

Hard to make a generalization about this

I don't think there is a need to switch to diazepam. we can taper on the benzo pt is on

Again, I am not sure about this. It feels a bit like guesswork or anecdotal at best.

Optional Comment/Feedback - Recommendation 5

Suggest we need to explain what ‘step wise’ means. Most clinicians do not understand what this is and why it’s important. Indeed I wonder if the reason why research on crossing over to a long-acting benzo is mixed is b/c a proper cross-over was not conducted. Will we also explain why clonazepam is problematic in terms of substitution?

Clonazepam for some has a longer inter-dose duration of action as diazepam has 2 short half-life metabolites.

This method works well for some. It needs to be individualized.

Switching from one BZD to another should be avoided as new substances may effect each patient differently. Providers should encourage patients to remain on their current BZD which may require more frequent dosing to reduce possibility of complications from using an alternative BZD.

I had no success tapering shorter acting Benzossuch as Alprazolam. Don't waste your time and the patient's time. Do a blended start with Diazepam ala Ashton.nt's time

---

I would not make the patient prove that they have inter-dose withdrawal before moving to diazepam. This choice could be part of the initial discussion with the patient, letting them decide whether to stick with the short-acting benzo or begin with a switch to diazepam.

I did find significant relief, an evening out of withdrawal/tolerance symptoms when I was crossed over from alprazolam to diazepam using Ashton's cross over schedule. While I know many have a hard time crossing to diazepam, I found it very beneficial.   That said, I do still experience interdose withdrawal even with my dosing diazepam 4x per 24 hour period, exactly every 6 hours.   I'm down to 8.00 mg diazepam, after being on 3.0 mg of alprazolam for decades.

Optional Comment/Feedback - Recommendation 6 9

Optional Comment/Feedback - Recommendation 6

Yes, and if the patient decides to abandon the taper, their decision should be respected..

"a previous dose" may need to be further defined

Pausing the taper or reverting back to a previous dose should occur as soon as possible if withdrawal symptoms are intolerable and/or if the patient requests this according to their need to stabilize their symptoms for improved functioning. Subsequent tapering should then be made more gradual.

---

these are general principles, some unique situations may require different approaches

---

Or can treat symptoms with alpha 2 agonists or anti epileptics until symptoms resolve and then recommence the taper and stop the adjuncts

---

This feels like it is unique to the patient. It is hard to assess what is the right approach as a generalized statement.

Might add ' some patient may choose to suspend the tapering process because of difficulties for long periods or indefinitely and this choice should be respected as the discontinuation process can be very unpleasant.'

Hold the line if possible

This in my opinion is an integral part of shared decision making.

Optional Comment/Feedback- Recommendation 7 7

Optional Comment/Feedback- Recommendation 7

"hyperbolic" not needed. "capability" instead of "ease"? By "coverage" do you mean "insurance coverage"?

Techniques or pharmaceutical formulations to facilitate hyperbolic tapering, including micro-tapering, should be presented to patients and/or caregivers on an individualized basis according to their ease of understanding, acceptability for safe administration, product availability, cost and/or coverage. (I agree w/ Mark's recommendation here to explain microtapering briefly) Note: I added tapering here alone - bc even people doing just cut-and-hold, for example, still need formulations to accommodate it.

Again, I am not convinced that hyperbolic micro-tapering is recommended.

Is 'hyperbolic micro-tapering' commonly used in the literature and practice? Is there evidence to support it as the 'best' way to taper? How does it compare to mini-tapering? Would it be better to say that patients should be provided with formulations that allow them to make small enough reductions in dose on whatever schedule keeps withdrawal symptoms tolerable?

Good to give examples of formulations- eg. manufacturers' liquids, compounded tablets/capsules/liquids. Define micro-tapering 'very small dose reductions made every day as compared to larger dose reductions made every two to four weeks which may reduce the impact of withdrawal'

The unfortunate reality for many patients is that this cost of compounded medication is often too high for them or not immediately accessable. Many plans within the US do not cover compounded medication or pharmacies refuse to provide compounding within their services. Cost and access in my opinion are the two biggest factors impacting patients.

? Define hyperbolic, micro-tapering

Optional Comment/Feedback - Recommendation 8 9

Optional Comment/Feedback - Recommendation 8

I would include "e.g., CBT"

Psychosocial interventions like CBT may not be effective for those severely harmed, as it resembles more of a brain injury than a psychological problem. I would support more practical assistance, such as help with filling out disability paperwork, accessing physical therapy, and addressing challenges with basic tasks like driving, cooking, and cleaning. Some insurance plans provide rides to appointments, and virtual appointments might be a more convenient option.

Which "psychosocial interventions" may be useful requires elucidation. CBT or sleep hygiene, for example, are not nearly as useful for managing withdrawal symptoms as some providers seem to believe. Lived experience is needed to weigh in on efficacy and desirability of various psychosocial interventions. More useful are things like in home health care, help with groceries and transportation, assistance navigating disability.

---

should we give examples? Or mention that success of different options may vary depending on the individual and/or the severity of their withdrawal.

But what if such interventions are neither available nor affordable?

'Acknowledging that gradual, hyperbolic tapering is likely to be the mainstay of safe tapering as psychosocial interventions are unlikely to be able to substitute for careful tapering to avoid intolerable withdrawal effects''

Psychosocial interventions are just as important as the pharmaceutical intervention. In fact this form of patient support is arguably more important but often comes back to both cost and access as the most identifiable barriers to care.

Agree in principle, but I worry that providers who can provide such psychosocial support are hard to find. I've never heard of therapists who provide this service.

Optional Comment/Feedback - Recommendation 8

It depends on whether the person providing the psychosocial help is knowledgeable in the symptoms and challenges of BZRA tapering. For example, most psychologists have no training in this subject and their advice could be harmful (i.e. suggesting that their client is suffering from some "underlying disorder", rather than recognizing the client is suffering from typical BZRA withdrawal symptoms). This could result in further harm. On the other hand, a peer support specialist with lived experience could be an incredible help, even save the person's life. If the psychosocial provider was knowledgeable in BZRA withdrawal, then I would strongly agree with this statement.

Optional Comment/Feedback - Recommendation 9 14

Optional Comment/Feedback - Recommendation 9

overall these seem supportive, but i would like to see more evidence

Some forums breed negativity and are not therapeutic.

Peer-support communities can be a forum for sharing helpful information; however, they can be harmful when they spread fear an misinformation. Careful monitoring by knowledgeable web administrators can minimize the spread of misinformation and provide helpful context to members' comments when necessary.

I would drop "communities" and make it stronger than "may be": "are often"

I strongly agree, but these community groups also have many downsides. People in these groups are often suicidal and overwhelmed. For decades, these peer support groups have served as a substitute for appropriate medical care and understanding. They might be more effective if the medical field had a better understanding of benzodiazepine harm, as this could help participants feel less distressed and overwhelmed.

Because tapering can be such a lengthy and intensive process, I suspect there will always be a role for peer support, and it would be to our benefit for these services to become more professionalized as well.

keyword "may"

Not sure how evidence based this truly is.

This needs to be tested more fully. It seems like a better approach than online support.

This is key

It depends on the peer support community and how it is managed. Some are helpful. Some are not. Would it be better to just say “Peer support may be helpful.”

There may be overwhelm of these communities. It would be better if there was a system set up in mainstream healthcare to avoid an overwhelming burden on these groups. If doctors are prescribing these drugs they should provide the means to stop them as well.

Peer-support is a pillar but again often hinges on individuals providing free or low cost access. Not enough people are willing to provide high quality low/no cost services and this also should not be the expectation. Services like this should have provider/insurance fee for service or value based care models to improve access to those most in need.

“Should be recommended “?

Optional Comment/Feedback- Recommendation 10 10

Optional Comment/Feedback- Recommendation 10

Add-on medications and supplements are extremely risky for those with impaired central nervous systems from benzodiazepine exposure.

Excellent point. Providers typically do not appreciate the prevalence of adverse effects in this population.

---

my general position, but this happens all the time

I routinely use adjuncts in my practice with very good results

---

I am not sure waht an adjuntive non-BZRA pharmacotherapy is.

Addition of other medications cannot substitute for adjusting a taper rate to suit an individual. It may complicate the picture by adding adverse effects and interactions. Many such drugs themselves cause physical dependence and will need to be tapered similarly to the benzodiazepine.

Flumazenil infusions should be considered under clinical trial governance and/or by experienced clinicians without conflict of interest. Some relatively harmless medications can be used as a segue to ceasing benzodiazepines, and also underlying conditions should ideally be treated. All treatment options should still remain on the table.

Adjunctive therapies are well studied and many patients do best with them.

This is highly dependent on the provider managing the tapering process. Although there is limited or low quality evidence to support adjunctive pharmacotherapies, that doesn't stop individual providers from looking past this and convincing patients that these adjunctive pharmacotherapies are benefitial.

I don't see much downside with offering routine second-generation antidepressants approved for anxiety disorders or offering prn's of meds like hydroxyzine. Barring cardiovascular contraindications, some patients may benefit from sympatholytics such as propranolol or clonidine, either routine or prn. Otherwise, the patient's anxiety may be exacerbated if they feel they have no backup/'failsafe' to take, adding to the anxiety caused by benzo w/d.
